# Markerless Motion Analysis Using New Digital Technology

**DOI:** 10.1298/ptr.R0037

**Published:** 2025-07-03

**Authors:** Megumi OTA

**Affiliations:** 1 Faculty of Health Sciences, Kyorin University, Japan

**Keywords:** Markerless, Sensorless, Motion analysis, Posture-tracking algorithm

## Abstract

Motion analysis is essential for physical therapists and athletic trainers to understand the motor function of their patients or athletes. Although marker-based motion analysis systems have been widely utilized in research, they are expensive and demand significant time and effort for measurement and analysis, which can complicate their application in clinical practice. In recent years, markerless motion analysis technologies have emerged as affordable and portable alternatives. These technologies include inertial measurement unit (IMU) sensors, depth cameras, manual digitization, and posture-tracking algorithms. IMU sensors detect motion using accelerometers and gyro sensors and can be worn on body parts. Depth cameras use infrared or laser technology to capture three-dimensional (3D) motion without requiring markers. Manual digitization enables semiautomatic identification of joint positions from images, allowing joint angle measurement without using specific cameras or markers. Posture-tracking algorithms use artificial intelligence to approximate joint positions from standard camera images, enabling automated motion analysis. Despite the enhanced accessibility of these technologies, limitations remain, particularly in analyzing detailed joint movements or individuals with structural abnormalities, and their accuracy depends on the environment and motion task. However, with further development, these technologies are expected to become increasingly reliable and provide physical therapists and athletic trainers with valuable, cost-effective, and easy-to-use tools for assessing movement in clinical and sports settings.

## Introduction

Physical therapists and athletic trainers often need to assess movement in clinical settings for patients^1–4)^ or various sports for athletes^5,6)^. This assessment aims to identify possible problems and abnormalities that may result in injuries^7)^. In rehabilitation and sports biomechanics, quantitative analysis of human body kinematics and kinetics has emerged as a potent instrument. This analytical approach has been used to review and improve athletic performance by improving technique^8)^, identifying injury risk factors^9)^, and supporting recovery from injury^10)^ or trauma^11)^.

Motion can be directly observed for motion analysis. However, the accuracy of the results depends on the evaluator’s skill and experience^7,12)^. Studies conducted over the years generally concur that the reliability of observation in this context is at best only moderate^12–15)^, and issues regarding validity and reliability persist in clinical practice^16)^.

For more detailed motion analysis based on objective data, numerous studies have consequently used marker-based motion analysis technologies, such as VICON and Optotrak^17–19)^. However, these technologies have certain disadvantages owing to the high costs of the equipment and time and technical skills requirement^12,20,21)^. Therefore, their use is largely limited to specialized environments such as hospitals, clinics, and laboratories. In most clinical and sports settings, creating an environment where marker-based motion analysis technologies can be used is complicated^22)^. Therefore, alternative systems that are portable, inexpensive, and can immediately provide objective data with a high degree of validity are required (Table 1).

**Table 1.** Disadvantages of traditional marker-based motion analysis systems and improvements required for systems to be widely useful in clinical practice

	Marker-based motion analysis systems	Requirements for clinical usefulness
Cost	High	Low
Specialist skills	Required	Not required
Tailored environment	Required	Not required
Marker or sensor	Required	Not required or minimal
Data analysis	Requires complicated procedures	Real-time or immediate; automatic or semi-automatic
Interpretation of the results	Requires specialized knowledge	Simple and intuitive

In recent years, several markerless motion analysis technologies have been developed. These include technologies that use sensors such as inertial measurement unit (IMU) sensors, depth cameras, manual digitization, and posture-tracking algorithms. Thus, this study aimed to review the advantages and disadvantages of markerless motion analysis systems and describe some of the current systems available.

## IMU Sensors

IMU sensors consist of accelerometers that detect translational movements and angular rate (gyro) sensors that detect rotational motion. More than one IMU sensor can be affixed to the body to measure multiple dimensions of movement. These sensors are lightweight, with wearable options for use on shoes and belts or as watches.

Several systematic reviews have assessed the reliability and validity of IMU sensors in measuring kinematic and spatiotemporal data of the upper limbs, lower limbs, and trunk during movements^23–25)^. Some reviews have focused on the application of wearable sensors for various purposes, such as the assessment of walking^26)^, general gait analysis, ^27)^ running^28)^, and movement disorders in Parkinson’s disease^29)^. However, their results varied, depending on the measurement methods, motion tasks, and parameters. For example, a systematic review identified that some reports suggested that the validity and reliability of spatiotemporal parameter variability and symmetry during walking and running were poor to moderate. However, the spatiotemporal parameters generally showed good to excellent validity and reliability^26)^. Another study revealed that the accuracy varied depending on the motion task or positioning of the sensors. Specifically, variations were greater in the transverse and rotational planes than in the frontal or sagittal planes between IMUs and marker-based motion analysis technologies^27)^. Variations also increased with more intense tasks, suggesting that these technologies may only be suitable for certain tasks or intensities of exercise or activity^27)^.

Validity and reliability may also vary with the positioning of the accelerometers, gyro sensors, or IMU sensors^29)^. Practical limitations certainly appear to be an issue: a review that examined the use of IMU sensors for measuring upper limb movements emphasized the importance of sensor positioning, calibration, and battery life^30)^. Therefore, the method of attachment and positioning of the IMU sensors exerts considerable influence on data accuracy, necessitating careful consideration when applied.

Compared with marker-based motion analysis systems, IMU sensors generally appear to have good reliability for practical gait analysis in a clinical setting^31)^. However, a systematic review and meta-analysis emphasized the importance of clinician-directed decision-making when using these sensors. This finding suggests that sensor-based systems should be viewed as a supplement to expert clinician input rather than a substitute, particularly due to the expertise required for the appropriate positioning of the IMU sensors.

## Depth Cameras

In recent years, markerless motion analysis technologies have significantly advanced, such as the development of RGB-D cameras, which have largely originated from gaming systems^32)^. An RGB-D camera integrates a conventional RGB (i.e., red, green, and blue) camera with a depth sensor component, capturing both color and depth information simultaneously^33)^. The multipoint distance to the sensor object can be calculated by measuring the time it takes for the infrared or laser beam emitted from a depth sensor to reflect off the object^34)^. A three-dimensional (3D) distance image can then be obtained from the combination of the object outline and depth data^35)^. The greatest advantage of using such a camera for motion analysis is that it eliminates the need for attaching markers or sensors to the subject’s body^36)^.

Several studies have used RGB-D cameras to measure joint angles and spatiotemporal parameters to monitor gait and joint angles during balance tasks^35–40)^. However, their findings varied with camera placement^35,38,41)^ and context^36,42)^, indicating the need for further investigation. For instance, when measuring the sagittal angles of the hip, knee, and ankle joints during gait, a side-view positioning of the camera provides high validity^37)^, and a front-view or 45° positioning gives lower validity^39)^. Different systems also had different validity at different camera angles^43)^. Gait velocity, stride length, and stride time were also measured more accurately from a side view^38)^. Conversely, step width, mediolateral and anteroposterior stability, and gait symmetry were more accurately captured from a front view^39)^. A front view was also more reliable for measuring the upper limb movements^44)^. Therefore, the placement of the camera should be adjusted to fit the target parameters and ensure that the key points to be estimated remain visible^22)^. However, a study found that the gait measurement errors using RGB-D cameras increased with increasing gait velocity^38)^. Therefore, motion analysis utilizing RGB-D cameras requires caution and may be limited to specific tasks or circumstances.

## Manual Digitization

Manual digitization refers to the manual localization of several joint centers in sequential images from different RGB camera perspectives. When multiple control points of known relative location are digitized in different camera views, the position of body points in the image can be reconstructed into real-space coordinates, most commonly through direct linear transformation^45)^. A primary benefit of manual digitization is that the attachment of markers is optional. This supported the development of this method to collect kinematic data, and the technique has been applied for many years^46)^. The use of a specialized camera, such as an RGB-D camera, is also not a prerequisite, and the analysis can use images from a standard RGB camera^46)^. However, this technique has some disadvantages, particularly that manual digitization is notoriously time-consuming, laborious, and prone to subjective errors^46)^. These limitations have encouraged the development of automatic solutions, facilitated by the emergence of more sophisticated technologies, such as artificial intelligence (AI).

## Posture-Tracking Algorithms

Over the past 10 years or so, automated vision-based motion analysis technologies have become more widespread. Similar to manual digitization, they use images captured by RGB cameras integrated into inexpensive and nonspecialized tablets and smartphones^47)^. Recent developments include the use of posture-tracking algorithms and AI-based technologies that can estimate body shape and joint positions from images and videos^48)^. This method relies on deep learning, utilizing algorithms that have been trained using extensive datasets of human body images^49)^. These algorithms can instantly and accurately detect the main joints and key points on the human body from various postures and angles, eliminating the need for markers or sensors, which makes them suitable for use in motion analysis^50)^.

In the early 2020s, studies on the validity of motion analysis using posture-tracking algorithms had increased^49–51)^. When comparing these algorithms with conventional marker-based motion analysis systems, the spatiotemporal parameters during gait demonstrated high validity^52,53)^. However, validity also depends on both the joint and motion task^54,55)^. For joint angles during gait, the validity is low for pelvic movements^56)^, and several studies have reported errors in the sagittal angle of the knee joint^52,56)^.

Various posture-tracking algorithms have been developed, such as OpenPose, DeepLabCut, TensorFlow MoveNet Lightning, and TensorFlow MoveNet Thunder. A study measured the sagittal angles of the hip and knee joints and spatiotemporal parameters during gait using several posture-tracking algorithms and a conventional marker-based motion analysis system and concluded that OpenPose was the most accurate for all parameters^57)^. This finding is consistent with the results of previous research, which reported that the differences between OpenPose and marker-based motion analysis were smaller than those observed for DeepLabCut^58)^. In contrast, another study compared OpenPose with DeepLabCut for the analysis of walking and found that the DeepLabCut Custom-trained model outperformed OpenPose^59)^. However, very few studies have compared motion analysis using DeepLabCut with marker-based motion analysis in humans to validate its accuracy. This makes it premature to draw definitive conclusions about the validity of DeepLabCut-based motion analysis^60–62)^. Similarly, no study has examined the validity of motion analysis using TensorFlow MoveNet Lightning or Thunder.

## OpenPose

OpenPose is a deep learning-based posture-tracking algorithm^63)^. Despite being relatively new, it has quickly become an important method for tracking human postures^49,50)^. This real-time technology detects body, foot, hand, and facial feature points (totaling 135 feature points) in a single image. This capability enables the recording of people moving using one or two digital video cameras, after which OpenPose is used to analyze trunk and limb movements from the captured images^63)^.

OpenPose is an open-source software included in the OpenCV library with a license for free noncommercial use^64)^. Commercial applications involve certain costs but may still be lower than conventional motion analysis systems such as the VICON motion system. In addition to the software, OpenPose image analysis only requires standard digital video cameras or commercially available digital camera-equipped tablets^63)^. Therefore, this technology is easy to use and portable and does not require a laboratory environment.

Researchers have been quick to recognize the potential use of OpenPose in clinical and sports settings. However, they have also been concerned that this potential depends on the accuracy of the analysis. Currently, validation of the accuracy of motion analysis using OpenPose in comparison with marker-based motion analysis is an active research area. Our investigations have established that OpenPose demonstrates reliability and validity in motion analysis during bilateral squats^50)^ and treadmill walking and running^49)^ in a healthy population.

At present, some nuances are associated with the use of this technology. First, the results for bilateral squatting showed very good test–retest reliability for OpenPose, and the test–retest reliability values for the sagittal angles of the trunk, hip, knee, and ankle joints were very similar between OpenPose and VICON^50)^. Conversely, proportional biases were noted in the sagittal angles of the trunk and hip joints measured. One reason is that VICON is a 3D motion analysis system, whereas OpenPose provides two-dimensional (2D) motion data from images captured by a single digital camera. In VICON, the trunk is defined as the sagittal displacement of the thoracic segments in global coordinates. Conversely, in OpenPose, it is defined as the sagittal plane angle of a straight line connecting the neck and center of the right and left hip joints relative to a line perpendicular to the floor passing through the vertical line. In VICON, the hip joint is defined as the sagittal plane angle of the thigh relative to the pelvis. On the contrary, in OpenPose, it refers to the sagittal plane angle of a straight line connecting the hip and knee joints relative to a straight line connecting the neck and center of the right and left hip joints. Therefore, the biases are more pronounced when trunk movements are associated with flexion or extension of the upper trunk relative to the lower trunk and when hip joint movements are associated with anterior or posterior pelvic tilt.

**Fig. 1.**
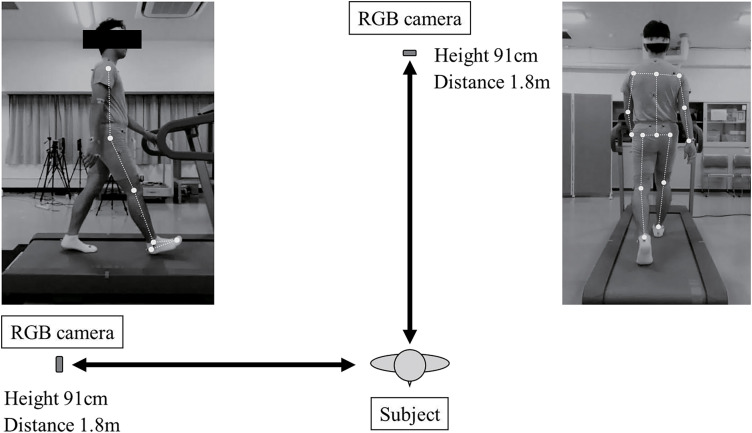
Experimental setup for motion analysis using a treadmill Digital video cameras were set 1.8 m behind and to the right of the subject. The height of the camera lens was set to 91.0 cm. Digital video cameras are popular RGB cameras that are built into commercially available tablets. Images captured from the back were measured at frontal angles, whereas those from the right were at sagittal angles^49)^.

**Fig. 2.**
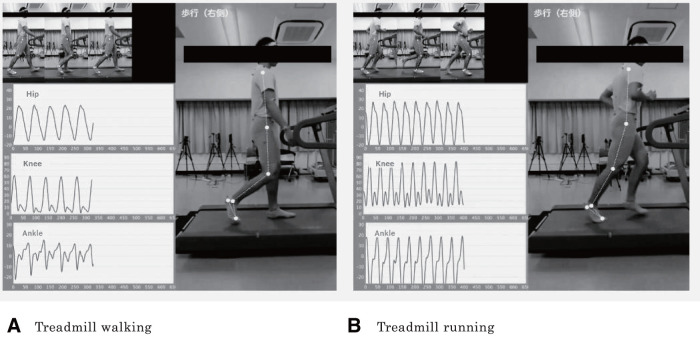
Typical example of temporal changes during gait The sagittal angles of the hip, knee, and ankle joints were measured by the motion analysis system using OpenPose during walking (A) and running (B). Gait velocities were set at 4 km/h for walking and 8 km/h for running^49)^.

When analyzing treadmill walking and running using OpenPose (Figs. 1 and 2), the algorithm demonstrated high validity for the range of motion and peak angles during the gait cycle in the sagittal plane of the knee and ankle joints, with no proportional biases^49)^. However, the peak angles during the gait cycle in the sagittal plane of the hip joint were influenced by the anterior or posterior pelvic tilt, which resulted in a proportional bias in the measurements. During the gait cycle, the range of motion in the sagittal plane of the hip joint demonstrated very high validity as long as the pelvic position remained stable, which did not significantly affect this measurement. Conversely, the range of motion and peak angles in the frontal plane of the pelvis and hip joint showed low validity, with both fixed and proportional biases.

When rotational motion of the trunk or hip joints, spinal flexion or extension, and anterior or posterior pelvic tilt are absent, a 2D analysis using OpenPose can be an adequate approach to motion analysis and provide valid results. Adjustments of the absolute values of the joint angles may facilitate the elimination of fixed biases, achieving more accurate measurements.

## Limitations of the Motion Analysis Using Posture-Tracking Algorithms

Several limitations of motion analysis technologies are associated with posture-tracking algorithms such as OpenPose. For example, errors between the true values indicating the actual joint movement and the data obtained using posture-tracking algorithms or a marker-based motion analysis system have not yet been fully explored. Verifying the clinical utility of posture-tracking algorithms will require correcting the data for fixed and proportional biases and investigating the error between the true values and the data obtained from the posture-tracking algorithm, including those with corrections. This must be performed across a range of activities and tasks and across groups or populations.

In addition, posture-tracking algorithms are 2D motion analysis technologies, so movements involving rotation may reduce their accuracy and therefore limit their applications^65)^. In recent years, methods that can produce 3D images by synchronizing multiple RGB cameras and processing images with posture-tracking algorithms have been developed. For example, studies that have explored the use of multimodal sensing technologies coupled with AI-based algorithms concluded that data obtained from more sensors may improve the effectiveness of AI models^66)^. However, this has economic and operational costs and is therefore a trade-off against the main advantage of the low cost and simplicity of the posture-tracking algorithms.

The use of posture-tracking algorithms to measure and analyze movement is also limited to the major joints. Some posture-tracking algorithms such as the Skinned Multi-Person Linear Model^67)^, Human Mesh Recovery, and Video Inference for Body Pose and Shape Estimation can be employed to measure movements of the upper trunk, lower trunk, and pelvis separately^67)^. However, the accuracy of these systems can still be improved. The movement of minor joints, such as those between vertebrae, can be measured accurately using a marker-based motion analysis system with more markers fixed directly to the skin surface^18)^. However, estimating their movements using posture-tracking algorithms is challenging.

Finally, most verification studies of motion analysis technologies using posture-tracking algorithms have been limited to young, healthy individuals. They have not included older people or patients with musculoskeletal structural abnormalities. For example, whether the knee joint position can be accurately estimated in patients with osteoarthritis presenting with varus deformity or whether abnormal motions, such as lateral thrust of the knee joint, can be measured is unclear. Therefore, further studies are needed to verify the clinical utility of posture-tracking algorithms among patients with specific conditions and older people more generally.

## Outlook for the Future

Minimizing the dependence on physical markers for motion analysis was demonstrated to facilitate data collection^48)^. Alternative technologies also have the potential to expand quantitative studies of human movement to situations in which marker-based motion analysis is not feasible or hinders research.

In the future, as these simple and inexpensive devices and systems become more widely available, objective measurements will be possible in hospitals, nursing homes, schools, workplaces, and sports settings. At present, whether joint points can be accurately estimated using posture-tracking algorithms in people with anatomical differences in body structures, such as amputations or joint deformities, is unclear. Moreover, whether these algorithms can assess abnormal movements that are only seen in certain patients, such as lateral thrust and collapse of the knee joint, remains to be clarified. The usefulness of this technology will increase further if its validity can be demonstrated for complex movements such as those in sports or activities of daily living, not simply basic movements such as gait and squatting. The optimal solution may involve utilizing the features of posture-tracking algorithms wherever feasible while being supplemented with other technologies as needed. For example, a study reported the attachment of multiple accelerometers from the head to the sacrum to measure acceleration and the instantaneous phase^68)^. If researchers opt to measure the inclination of the spinal column using IMU sensors while assessing major joints with posture-tracking algorithms, the potential applications could be further expanded.

The use of these technologies across various settings is likely to facilitate the acquisition of substantial kinematic and kinetic data from a wide range of individuals, including healthy people, patients, children, older people, and athletes. In clinical settings, it would be extremely valuable to be able to rapidly and immediately assess the motor function of individuals, record objective and accurate data over time, and provide feedback, as needed. Analyzing this comprehensive dataset could help estimate and reduce the risk of developing musculoskeletal disorders and falls. The advent of more efficient data analysis technologies, which reduce both time and cost, will streamline the evaluation of motor functions through vision-based motion analysis using individuals’ personal smartphones or tablets. This technology is also expected to be useful in applications that provide individuals with personalized exercise programs and track their progress.

Markerless motion analysis technologies have yet to achieve widespread implementation within the field of biomechanics. The commercial sector as yet offers a limited selection of technologies. However, it is evident that this technology is developing rapidly. The advent of advanced posture-tracking algorithms will only enhance the robustness, flexibility, and accuracy of markerless systems and make them more useful for physical therapists and athletic trainers.

## Conclusions

The technology for vision-based motion analysis is still developing^46)^. However, significant improvements have been made in the flexibility and accessibility of motion-tracking technologies. Accuracy, particularly in joint rotation and movement complexity, is still limited. Nevertheless, the rapid evolution of posture-tracking algorithms such as OpenPose shows great promise. With further validation studies, particularly in the context of clinical and complex movements, these technologies could become invaluable tools for both physical therapists and researchers. Therefore, to develop more comprehensive motion analysis solutions, future developments should focus on reducing bias in data, improving accuracy in non-ideal conditions, and integrating these systems with complementary technologies, such as wearable sensors.

## Acknowledgments

I would like to express my deepest gratitude to Professor Ichihashi Noriaki of Kansai Medical University and Professor Tateuchi Hiroshige of Kyoto University for their invaluable participation in my studies.

## Funding

Our studies^49,50)^ presented in this paper received research funding from Smart Health, a subsidiary of the MIXI Group.

## Conflicts of Interest

The studies^49,50)^ presented were conducted in collaboration with Smart Health, which is part of the MIXI Group. During the period when the studies were conducted, I was affiliated with a collaborative research laboratory at Kyoto University established through a donation from Smart Health.
